# Prediction and detection of virtual reality induced cybersickness: a spiking neural network approach using spatiotemporal EEG brain data and heart rate variability

**DOI:** 10.1186/s40708-023-00192-w

**Published:** 2023-07-12

**Authors:** Alexander Hui Xiang Yang, Nikola Kirilov Kasabov, Yusuf Ozgur Cakmak

**Affiliations:** 1 Cakmak Lab, Department of Anatomy, University of Otago, Dunedin, New Zealand; 2 School of Engineering, Computing and Mathematical Sciences, Auckland University of Technology, St Paul street, AUT, Auckland, 1010 New Zealand; 3 George Moore Chair of Data Analytics, Ulster University, Londonderry, UK; 4 Institute for Information & Communication Technologies, Bulgarian Academy of Sciences, ul. Acad Bonchev, 2, Sofia, 1113 Bulgaria; 5 Medtech Core NZ, Auckland, New Zealand; 6 Brain Health Research Centre, Dunedin, New Zealand; 7 Centre for Health Systems and Technology, Dunedin, New Zealand

**Keywords:** Cybersickness, Detection, Prediction, Biometrics, Physiological, Machine learning, AI, Neural networks, Virtual reality, Extended reality, Simulator, EEG, ECG, HRV, Spiking neural network, Brain, Dynamics, Spatiotemporal, NeuCube

## Abstract

Virtual Reality (VR) allows users to interact with 3D immersive environments and has the potential to be a key technology across many domain applications, including access to a future metaverse. Yet, consumer adoption of VR technology is limited by cybersickness (CS)—a debilitating sensation accompanied by a cluster of symptoms, including nausea, oculomotor issues and dizziness. A leading problem is the lack of automated objective tools to predict or detect CS in individuals, which can then be used for resistance training, timely warning systems or clinical intervention. This paper explores the spatiotemporal brain dynamics and heart rate variability involved in cybersickness and uses this information to both predict and detect CS episodes. The present study applies deep learning of EEG in a spiking neural network (SNN) architecture to predict CS prior to using VR (85.9%, F7) and detect it (76.6%, FP1, Cz). ECG-derived sympathetic heart rate variability (HRV) parameters can be used for both prediction (74.2%) and detection (72.6%) but at a lower accuracy than EEG. Multimodal data fusion of EEG and sympathetic HRV does not change this accuracy compared to ECG alone. The study found that Cz (premotor and supplementary motor cortex) and O2 (primary visual cortex) are key hubs in functionally connected networks associated with both CS events and susceptibility to CS. F7 is also suggested as a key area involved in integrating information and implementing responses to incongruent environments that induce cybersickness. Consequently, Cz, O2 and F7 are presented here as promising targets for intervention.

## Introduction

Virtual Reality (VR) technology is becoming prevalent in entertainment, art, education, social and professional settings [1, 2]. VR allows for interactive immersion into shared digital environments that can be accessed by many. Despite this, individual experiences in VR remain far from idyllic. Drawbacks exist in the form of cybersickness (CS)—a debilitating sensation accompanied by a cluster of symptoms that include nausea, oculomotor issues and dizziness [3]. It is unfortunate that limitations to human physiology and perception form a barrier to consumer adoption of VR technology; especially since our world continually charges towards a nexus of virtual and real-world interactions. A way to combat CS would be to utilize a tool that predicts or detects it. Yet, these tools must be automated and objective, so that preparations or active responses like training resistance, timely warning systems and clinical intervention can be implemented. Tracking of CS is currently restricted to subjective reports through verbal confirmation or questionnaires. Not only do these methods not allow for future prediction, but they are time inefficient and require manual input. With current technology at our disposal, objective biomarkers correlated with cybersickness can be collected from wearable devices and fed into machine learning algorithms for streamlined, automatic prediction and/or detection of cybersickness events [4]. While various models for prediction and detection of CS severity have been proposed [4, 5], there lacks a way to both collect CS data and continue to generate new knowledge about the condition through machine learning assisted approaches. To achieve this, the present study uses a modified version of the brain-inspired NeuCube spiking neural network (SNN) architecture [6] to both predict and detect CS whilst generating new knowledge about the condition.

There are several reasons for choosing SNNs for this purpose. SNNs are advanced machine learning techniques [7] and are considered the third generation of artificial neural networks. They simulate the behaviour of biological neural networks by creating and updating connections between spiking neurons (synaptic connections) to learn temporal associations between them. This architecture and mechanism of learning has several advantages in temporal information processing [8-13] over that of traditional neural networks. This includes robustness to noise through the encoding of consecutive time series data, such as EEG, into a compressed data format known as spikes (binary units) [7]. Encoding procedures such as threshold-based-spike-generation, produce spikes that represent a change in consecutive values above a certain threshold, allowing for changes in data to be captured over time. Additionally, if multiple time series, such as EEG channels, are modelled in a single SNN, patterns of interactions between the changes in their time series can be detected and analyzed. SNN architectures can further benefit from the usage of brain templates that specify a spatial distribution in the anatomical shape of a brain. Upon training, these models can be considered an interpretable spatiotemporal map of the brain activities measured, which assists to better understand brain dynamics under diverse conditions. Further on, this spatiotemporal map can be represented as a feature vector, and additional parameters from other biologically relevant data such as HRV can be added for classification of different brain states.

Consequently, the present study performs deep learning of integrated EEG and sympathetic heart rate variability (HRV) data in an interpretable dynamically evolving SNN architecture. This architecture mimics the biological structure and processing mechanisms of the human brain, and captures spatiotemporal information from EEG signals to form a dynamically updateable neural map of CS. A machine learning algorithm was developed that can detect CS events (76.6%) and predict it prior to VR usage at resting baseline (85.9%) using electroencephalogram (EEG) data. F7 alone was the most optimal input for cybersickness prediction. The algorithm also integrated fusion of electrocardiogram (ECG) heart rate variability data but it did not improve classification accuracy. The study found that features related to cybersickness susceptibility are diverse and that highlighted features change over time. Amongst many important features, Cz (premotor and supplementary motor cortex) and O2 (primary visual cortex) are key hubs in functionally connected networks associated with both CS events and susceptibility to CS. According to accuracy results and analysis of CS related brain hubs, Cz, O2 and F7 present as promising targets for intervention. The study additionally proceeded with an exhaustive analysis to find the best time segment during a resting-state EEG baseline and its data length for optimal prediction accuracy.

## Contributions

In summary, the paper contributes the following:

- A novel approach to the prediction and detection of cybersickness using interpretable spiking neural networks (SNN) and weighted K-nearest neighbor (KNN) algorithms using EEG and ECG data, both separately and in their integration.
- Optimized SNN architecture based on inherent characteristics of cybersickness
- Machine learning assisted knowledge discovery and insight into the spatiotemporal brain dynamics of cybersickness
- Considerations for feature reduction for diagnostic and predictive CS computational models.
- Machine learning extracted clinical biomarkers for the development of intervention strategies.

## Methods

### Subjects

Sixty-four participants, male (29) and female (35), age range of 18–33 years (mean 23, standard deviation ± 4.1) were recruited from the student and working population. The exclusion criteria were a previous diagnosis of neurological disorder, cardiovascular disease, diabetes, gastrointestinal disorder, medications, or smoking. All subjects had either normal or corrected visual acuity with contact lenses. This study was approved by the University of Otago Ethics Committee (H20/169) and performed in accordance with relevant guidelines and regulations. All participants provided signed consent.

### Experimental equipment

A VR video of rotating stars published by previous researchers was played in an HTC Vive headset (HTC Corporation, Taipei, Taiwan) [14]. EEG was recorded using starstim32 (Neuroelectrics). ECG was recorded using Shimmer3 5 lead ECG (Shimmer, Dublin, Ireland) at a sampling rate of 512 Hz. Five electrodes were placed, two 5 cm above the pelvic girdle, labelled according to proximity towards the left leg (LL) and right leg (RL), and two 5 cm below the clavicle, labelled according to proximity towards the left arm (LA) and right arm (RA), with the fifth electrode at the V3 position relating to the midway point between the 4th and 5th intercostal space. Data obtained from the LL-RA channel between electrodes were used for analysis.

### Software

iMotions 8.0 (iMotions, Cophenhagen, Denmark) was used to synchronize EEG and ECG data recordings for a unified collection of measurement time series. Live view of biosensor data streaming ensured quality data collection and so that markers separating baseline, stimulation and post stimulation could be placed during the experiment. Neucube was used for the SNN architecture and feature vector production. Python 3.8.8 was used for the classification algorithms and neuron proportion visualisation. HRV was analyzed using Kubios HRV Premium Ver. 3.3 software [15] (Kubios, Kuopio, Eastern Finland). For 10 s HRV results, Neurokit2 [16] was used to determine R-peaks and pyHRV [17] was used to calculate RMSSD. BrainNet Viewer was used to visualize feature interaction networks [18]. The VR video used in this experiment was developed in previous work by researchers from Stanford University, and was chosen for its propensity to induce cybersickness in individuals. The VR video consists of clockwise rotating white dots about the roll axis, dispersed at different depths through the visual foreground and background [14].

### Protocol

**Fig. 1:**
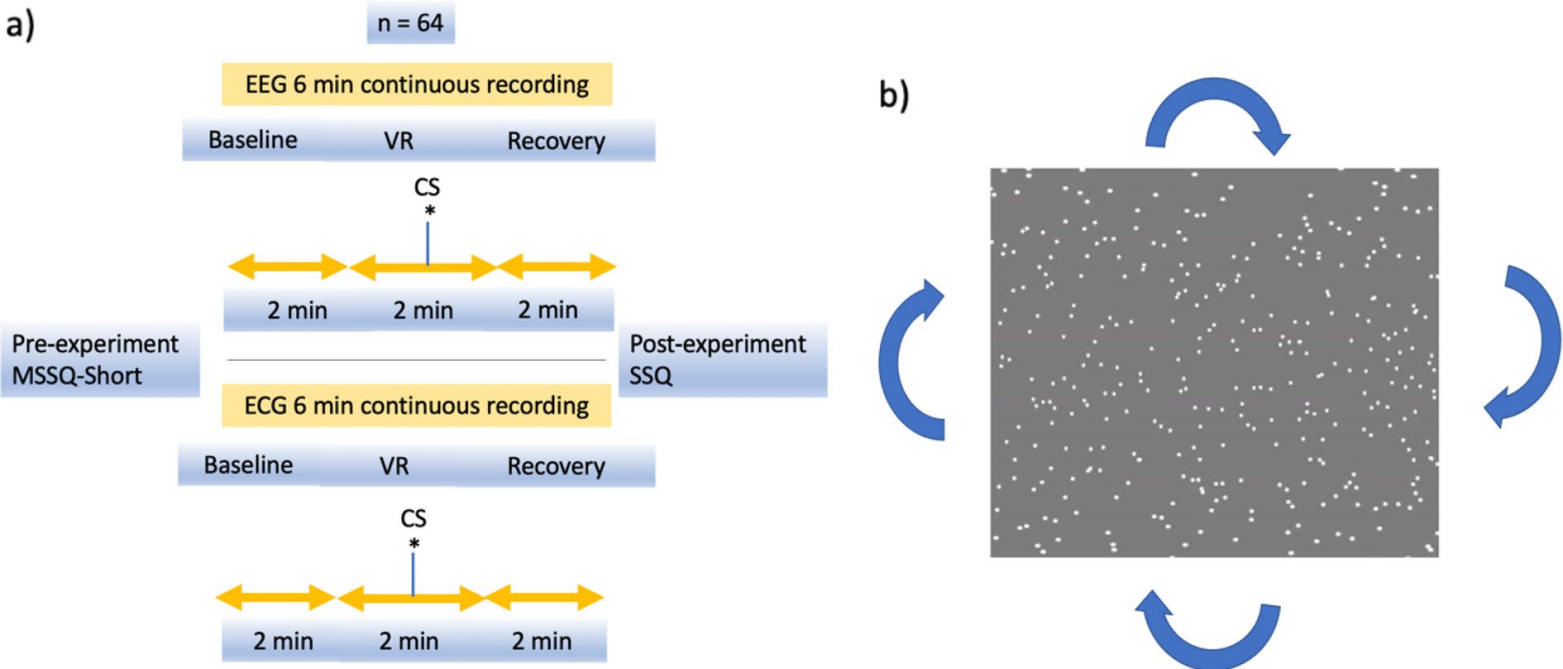
**a** Experiment flow, **b** VR video example

Participants (*n* = 64) underwent a 2 min resting state baseline (A) before VR immersion without HMD usage, then watched a 2 min VR video of rotating stars (B)_,_ followed by removal of the headset and a 2 min recovery period. EEG and ECG was recorded continuously throughout the entire experiment. To mitigate any potential noise, participant immersion in VR was a passive ordeal where the only requirement was to stare straight ahead with minimal body and head movement, and all parts the experiment were seated. The conscious perception of cybersickness was reported via a thumbs up, and was simultaneously marked on the data stream. Individuals who reported cybersickness and those that did not (controls) were separated into two groups. A pre-experiment motion sickness susceptibility questionnaire [19] (MSSQ-Short) was administered to assess motion sickness history and susceptibility, along with a post-experiment simulator sickness questionnaire [20] (SSQ) to collect individual sickness ratings Fig. 1.

### Statistics

A Mann–Whitney U test was run to compare between cybersick and control groups for the following data: MSSQ-short scores, SSQ scores, spike count and HRV parameters—Parasympathetic nervous system index (PNS), sympathetic nervous system index (SNS), stress index (SI), standard deviation of N–N intervals (SDNN), root mean squared of successive differences of R–R intervals (RMSSD).

### *The NeuCube brain-inspired spiking neural network architecture*

**Fig. 2:**
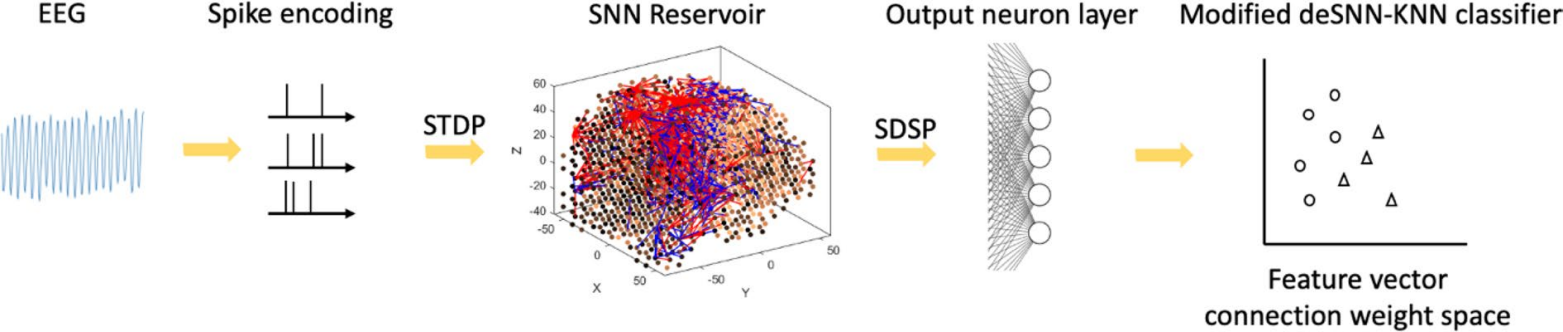
Data pipeline. (spike timing dependent plasticity) STDP, (spike driven synaptic plasticity) SDSP, (dynamic evolving spiking neural network-k nearest neighbour algorithm) deSNN-KNN

The following sections below describe the general architecture of the model and data pipeline. This includes initial encoding of the raw EEG data into spikes, training of the SNN reservoir for knowledge discovery and feature selection, producing a feature vector which represents the spiking activity in the neural network through connections with an output neuron layer, and finally classification of this feature vector. A graphical representation of this data pipeline is shown in Fig. 2.

### Spike encoding

SNNs receive reconstructed input signals as binary waveforms known as spike trains. Thus, the raw EEG data must first be transformed into this format. Step Forward (SF) encoding was used as a ‘signal to spike encoder’. SF is a threshold-based algorithm that works based on updating cutoff values for excitatory and inhibitory spikes, according to a $\mathrm{base}$ value at time $t=0$ and a user defined threshold value. If a signal’s value is greater than the current excitatory cutoff $(\mathrm{base}+\mathrm{threshold})$ then an excitatory spike is encoded, and the excitatory cutoff value is updated as the new $\mathrm{base}$ value. If the signal’s value at $t$ is less than the current inhibitory cutoff value $(\mathrm{base}+\mathrm{threshold})$ then an inhibitory spike is encoded and the inhibitory cutoff value is updated as the new base value. In some cases, no spike is encoded and the base value remains the same [21]. Spike counts for every channel were extracted and compared between CS and control groups at baseline (A) and during the CS onset event (B).

### NeuCube reservoir

A reservoir of connected neurons was initialized in preparation for spike inputs. A SNN reservoir (SNNr) module is in principle scalable in size, and here it is composed of 1471 LIF neurons representing 1cm^3^ of the brain, located at the same coordinates as those modelled in the Talairach atlas to create a 3D-brain geometry. Defining the spatial location of neurons allows spatial–temporal patterns to be elucidated from spike inputs. Connection weights between reservoir neurons were randomly initialized using the small world connectivity (SWC) approach. The SWC limits connections to only form within a defined radius and the random connections creates a diverse set of dynamical states. Connection weights, also known as ‘synaptic weights’, modulate any increase or decrease in the membrane potential of the post-synaptic neuron. In other words, it is a measure of the contribution of a pre-synaptic neuron towards the firing of a post-synaptic neuron. Connections also hold an intrinsic value of ‘synaptic delay’, which is the time delay in firing between pre and post synaptic neurons. Excitatory and inhibitory synapses within the reservoir are probabilistically determined according to the following formula:

$$P_{i,j} = \left\{ {\begin{array}{*{20}ll} {C*e^{{ - \left( {{\text{d}}_{i,j}^{{{\text{norm}}}} /\lambda } \right)^{2} }} \quad if {\text{d}}_{i,j}^{{{\text{norm}}}} \le d_{{{\text{thresh}}}} } \\ {0 \quad {\text{otherwise}}} \\ \end{array} } \right.$$

where ${P}_{i,j}$ is the probability of establishing a connection between two neurons *i* and *j*; $C$ is the maximum connection probability; λ is the small world connection radius; ${d}_{i,j}^{\mathrm{norm}}$ is the normalized distance between two neurons; ${d}_{\mathrm{thresh}}$ is the maximum connection distance between two neurons. In this way, closer neurons have a higher probability of stronger connection weights than neurons further away.

### SNNr training

Training the SNNr involved unsupervised learning of spike trains introduced by ‘input neurons’ at 32 EEG channel locations. These locations were gained from the conversion of 10–10 scalp electrode positions into Talairach coordinates. Input neurons feed spike trains of each sample to the SNNr in a temporally synced and spatially distributed manner. Similar to the notion of summation at an axon hillock [22, 23], an output spike is produced by a post synaptic neuron when many input spikes from pre synaptic neurons accumulate over a short period of time. As spike trains spread throughout the SNNr, connection weights between reservoir neurons are updated according to a rule called ‘Spike Timing Dependent Plasticity’ (STDP). This sort of learning mimics cellular processes of long-term potentiation and long-term depression involved in learning and memory [24].

$$W\left( s \right) = \left\{ {\begin{array}{*{20}ll} {A_{ + } {\text{exp}}\left[ {s/t_{ + } } \right] \quad {\text{for}} \; s < 0} \\ {A_{ - } {\text{exp}}\left[ { - s/t_{ + } } \right] \quad {\text{for}} \; s > 0} \\ \end{array} } \right.$$

S is the time delay between presynaptic and post-synaptic firing. ${t}_{+}$ is the pre-synaptic time interval. ${t}_{+}$ is the post-synaptic time interval. ${A}_{+}$ is the amplitude of weight increase. ${A}_{-}$ is the amplitude of weight decrease.

The STDP rule implements a form of logical causality, in which connection weights increase or decrease proportional to the synaptic delay. If a presynaptic neuron fires before a post-synaptic neuron, the connection weight increases between them. Likewise, connection weights decrease if a postsynaptic neuron fires before a presynaptic neuron. The end product is a trained ‘SNNr cube’—a neuronal model with connection weights that represent complex and dynamic spatiotemporal brain activity.

In our study, the training samples were divided into two groups equally, CS (*n* = 32) and control (*n* = 32). A SNNr cube was trained on all 32 channels of EEG data for each group, giving two distinct SNNr cubes with different connection weights. The connection weights of these cubes were subtracted from each other, producing an SNNr cube specific to cybersickness.

### Knowledge discovery

Subtracted SNNr cubes were made using data 2 s in length selected from time segments 30–32 s and 90–92 s at baseline, and from 1 s before the CS event. Since connections between neurons at SNNr initialization are randomly generated, the same initialized connections were kept constant for subtractions between cybersick and control groups. Underpinning this subtraction, was the hypothesis that there would be different brain information processes and network dynamics in CS versus control subjects. In theory, these differences would not just appear during the manifestation of CS but also during resting-state baseline as a precursor to CS or marker of susceptibility. The reason behind selecting two time points at baseline was to see if these markers might change over time.

**Fig. 3:**
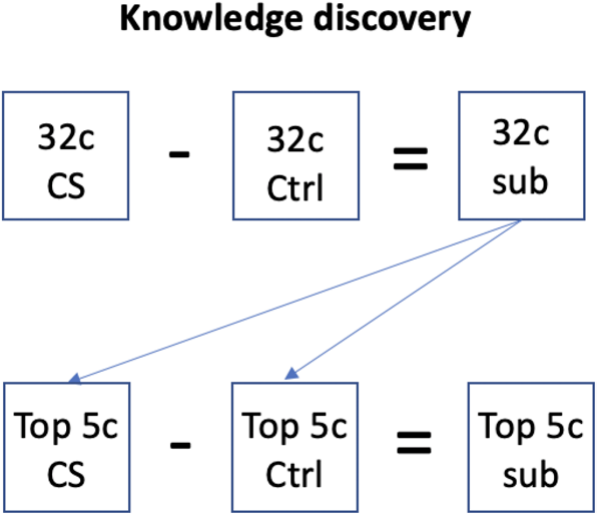
Finding the top five features to create a new SNNr cube with key CS information; *c* EEG channels, *CS *cybersickness, Ctrl Control, *sub* subtracted cube

Using the subtracted SNNr cube, clusters of reservoir neurons surrounding each input neuron were grouped by connection weight. Neuron proportion was calculated as the percentage of neurons in the cube belonging to each cluster. Total input cluster interactions were compared to each other in a Feature Interaction Network (FIN) analysis. FIN revealed relative strengths of functionally connected areas of the brain that discriminate between the two classes. The top 5 features (channels) by neuron proportion were chosen as input neurons to train a new SNNr cube, representing only the most informative features that define CS. Data for the control group during VR immersion were selected as the median time of CS induction, which was at the 39 s mark. This process is detailed in Fig. 3.

### Producing a feature vector

Default NeuCube processing uses one reservoir cube trained on all data samples for classification, with the notion that data of a certain label will have different spike activity and spike propagation than data of another label [25]. This study took a different approach by subtracting individually trained SNNr cubes, to produce synaptic connection weights within the reservoir that form a neural map specific to CS. This map is a template through which new data samples are parsed to obtain a feature vector, which is the synaptic connection weights between input + reservoir neurons, and output neurons. A dynamic evolving SNN algorithm (deSNN) was used to learn the association between class labels and the training samples in a supervised manner. deSNN has the advantage over other SNN classification models in that it is computationally inexpensive and boosts the importance of the order in which input spikes arrives, along with considering all other incoming spikes. Thus, it is suitable for on-line learning and early prediction of temporal events. In this algorithm, a new output neuron ($O$) for each training sample was created. These output neurons connect to every input and reservoir neuron ($N$). The connections have initial weights that are set according to the Rank-Order learning rule (RO).

$$w_{{{\text{init}}}} \left( {N_{n} ,O_{m} } \right) = {\text{mod}}^{{{\text{order}}\left( {N_{n} ,O_{m} } \right)}}$$

The RO learning rule boosts the importance of the first incoming spikes on neuronal synapses. The advantage of RO is fast, one-pass learning and asynchronous data entry of synaptic inputs. The value of the *mod* parameter for part 1 of this study was set to a default of 0.9. The *O*–*N *connection weights between the SNNr and the output deSNN neurons are then further dynamically tuned by the following spikes via spike driven synaptic plasticity (SDSP)—a modified version of STDP. Due to a bi-stability drift in the SDSP rule, once a weight reaches the defined high value (resulting in LTP) or low value (resulting in LTD), it is fixed for the rest of the training phase. The rate at which a weight reaches LTD or LTP depends on the values of the set drift parameter.

$$w_{{{\text{final}}}} \left( {N_{n} ,O_{m} } \right) = \begin{array}{*{20}c} {w_{{{\text{init}}}} \left( {N_{n} ,O_{m} } \right) + {\text{drift}}_{{{\text{up}}}} * n_{{{\text{spikes}}}} } \\ { - {\text{drift}}_{{{\text{down}}}} * n_{{{\text{spikes}}}} } \\ \end{array}$$

${\mathrm{drift}}_{up}$ is the value increase in synaptic weight after pre-synaptic firing. ${\mathrm{drift}}_{down}$ is the value decrease in synaptic weight with no pre-synaptic firing. ${\mathrm{drift}}_{up}$ is set to 0.08 and ${\mathrm{drift}}_{down}$ is set to 0.08 for part 1 of the study. SDSP works similar to STDP except that the post-synaptic membrane potential is assumed to always reach above threshold when the pre-synaptic neuron fires, leading to an increase in connection weight of the synapse between two neurons. At the same time, if no firing occurs from the pre-synaptic neuron, the connection weight of the synapse is decreased.

Altogether, the deSNN algorithm provided brain-inspired feature vectors for every sample, consisting of both input–output neuron connections, and reservoir-output neuron connections that can be classified.

The following connection strategies between the SNNr neurons and the deSNN classifier neurons were explored in this paper while searching for an optimal model:

- SNNr cube trained on all data of 32 input neurons; 1471 SNNr neurons connected to each output neuron in the evolved deSNN classifier;
- SNNr cube trained on all data of 32 input neurons; only the 32 input neurons are connected to the output neurons;
- SNNr cube trained on 5 channel data; 1471 SNNr neurons connected to each output neuron in the evolved deSNN classifier
- SNNr cube trained on all data using all combinations of 5 top input neurons (e.g. top channels); only the 5 input neurons are connected to each output neuron;

### ECG

The following heart rate variability parameters were computed: PNS, SNS, SI, SDNN, RMSSD. The selected time segments were 2 min, 30 s and 10 s in length. Only RMSSD was analyzed for the 10 s time segments, due to the statistical unreliability of the other parameters for this length of data. RMSSD is considered a reliable indicator for parasympathetic cardiac activity robust to the signal noise of respiration. Meanwhile, SI is an index for sympathetic activity. Both parasympathetic and sympathetic activity contribute to SDNN. PNS and SNS are validated indicators of parasympathetic and sympathetic activity [15, 26].

### Data Fusion

This study approached data fusion by combining feature vectors representing synaptic connection weights with the output layer of NeuCube and HRV variables that yielded the best accuracies. These include the best combination of parasympathetic or sympathetic features which would be added on to the final feature vector.

### Classification

Three different algorithms were used to classify the feature vectors, with leave-one-out cross validation (LOOCV):

### Modified KNN

A distance-based algorithm between data points. The study employed a modified version of KNN, in which the following parameters were optimized using an exhaustive grid search:

1. *k* is for all neighbours or restricted by class label;
2. Using Manhattan distance or Euclidean distance;
3. Distance initially weighted uniformly or by signal-to-noise ratio (SNR) that identifies the importance of the features (see the wwkNN method [38]);
4. Neighbours weighted during voting –
  - Uniform (equally)
  - By the inverse of their distance
  - By the function: $\frac{{{\text{max distance}} - \left( {{\text{neighbour}} - {\text{minimum distance}}} \right)}}{{{\text{max distance}}}}$
5. Feature weights weighted during voting –
  - Uniform for each feature
  - SNR for each feature

### Linear discriminant analysis (LDA)

An algorithm that finds linear combinations of features that separate classes along a hyperplane. Least squares solution was used with optimized shrinkage.

### Light gradient boosting machine (LightGBM)

LightGBM is a gradient boosting framework that uses tree-based learning algorithms.

Optimized for number of trees, learning rate, boosting type (gradient boosting decision tree, GBDT), gradient-based one-side sampling (goss), dropouts meet multiple additive regression trees (dart).

### Part 2: Algorithm Optimisation with Extensive Time Segment Analysis

Part 2 of the study used high capacity computing provided by New Zealand eScience Infrastructure (NeSI) to extend the previous analysis using the modified KNN algorithm. The goal was to find the best time segment for prediction out of the 2 min EEG resting-state baseline, partitioned into varying data lengths (2 s, 5 s, 10 s). The analysis similarly scans through all types of model training, and feature vector type in terms of connections to the output neurons as in Part 1. The difference is that *mod* and *drift* parameters for SDSP were optimized for classification of the best time segment for prediction and also for detection to see if this would improve accuracy. Additionally, the value of ${drift}_{up}$ was set to always be more than ${\mathrm{drift}}_{down}$, which implements stronger I–O connection increases compared to decreases. This type of SDSP also maintains stronger I–O connections for input neurons that fire more compared to those that fire less, thereby boosting their importance further. Varying data lengths serve to explore whether capturing more EEG data improves prediction accuracy, whereas optimizing for SDSP improves the transformation of the EEG data into the feature vector for classification.

## Results

### Part 1

#### MSSQ-short and SSQ scores

**Fig. 4:**
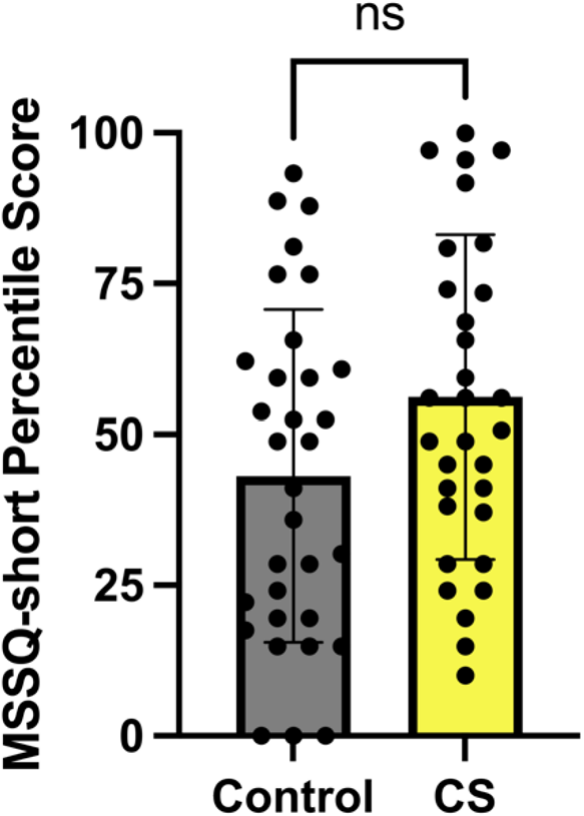
MSSQ-short scores *P* > 0.05. Error bars show ± SEM

**Fig. 5:**
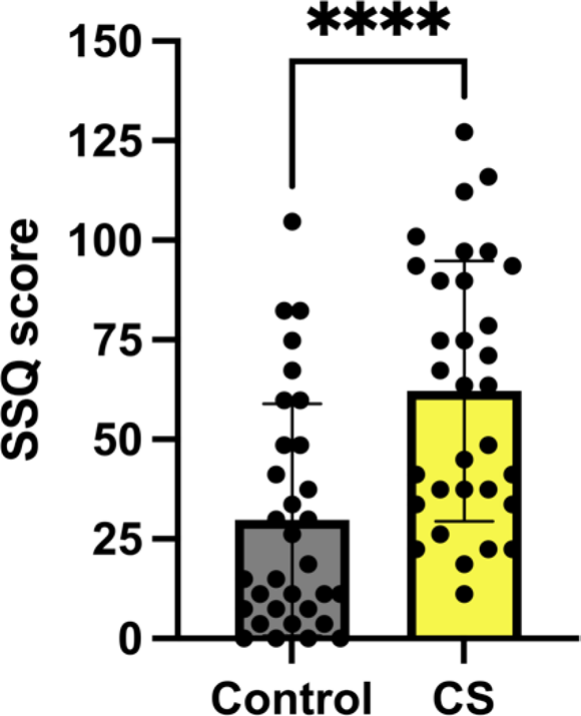
SSQ scores, **** = *p*  < 0.0001. Error bars show ± SEM

MSSQ-short scores did not differ significantly (*P* > 0.05) between CS and Control groups (Fig. 4). SSQ scores differed significantly between CS and control (*P* > 0.0001) (Fig. 5). CS groups had significantly higher SSQ scores than controls, showing that MSSQ-short percentile scores were not a good indicator of sickness in VR usage.

#### EEG

**Fig. 6:**
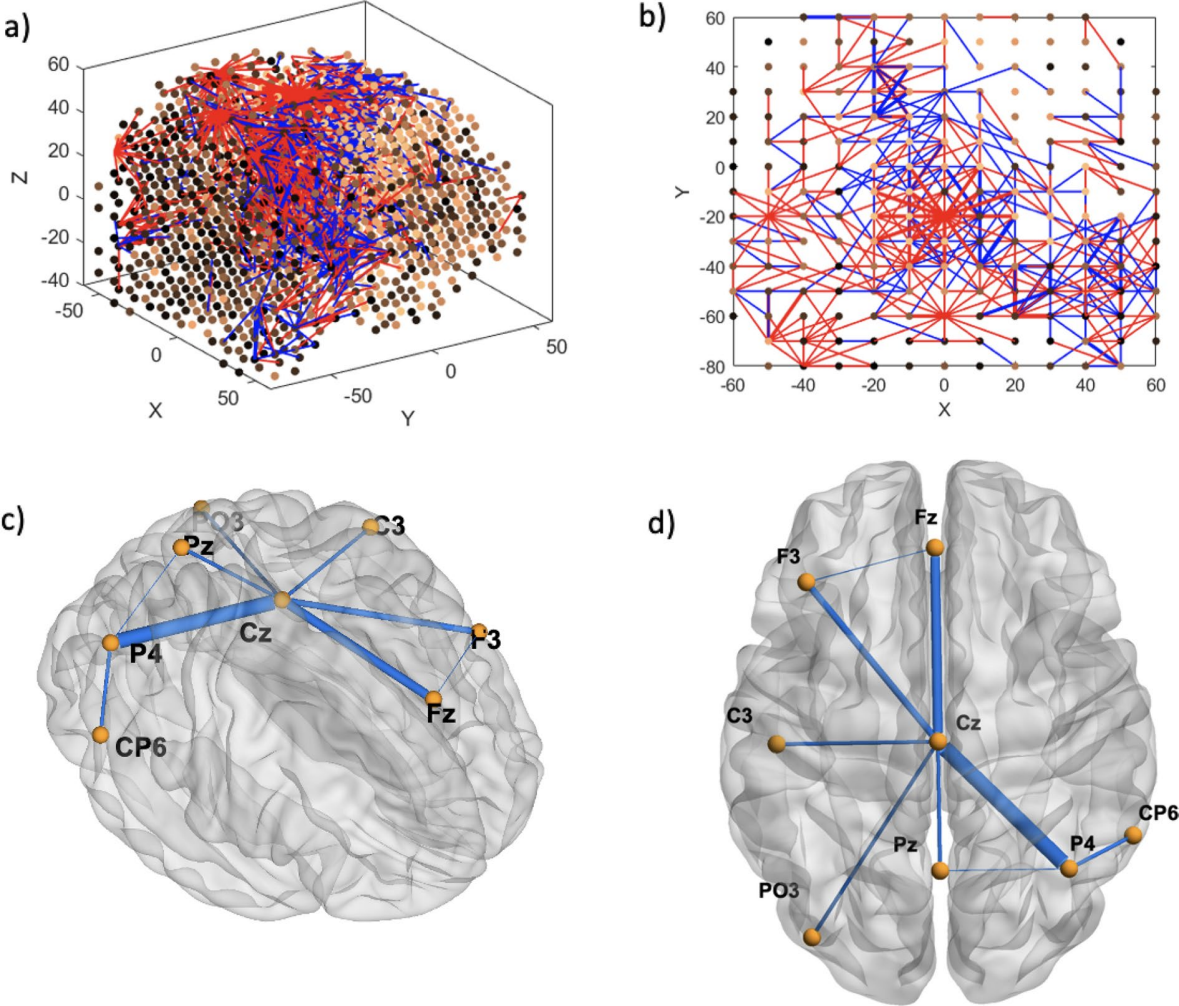
Resting-state baseline 30–32 s subtracted network dynamics. Functional connectivity of neurons in the SNNr is represented by **a** right hemisphere medial view of the SNNr and **b** axial view. Blue lines are positive connections, red lines are negative connections. Brighter neurons have stronger connections. Feature interaction networks between channels are represented by **c** right hemisphere medial view and **d** axial view. Thicker lines indicate stronger interaction whether they be positive or negative. These interactions confirm our hypothesis that even at baseline of 30–32 s, there is a significant difference between the brain information processes of CS versus control subjects

**Fig. 7:**
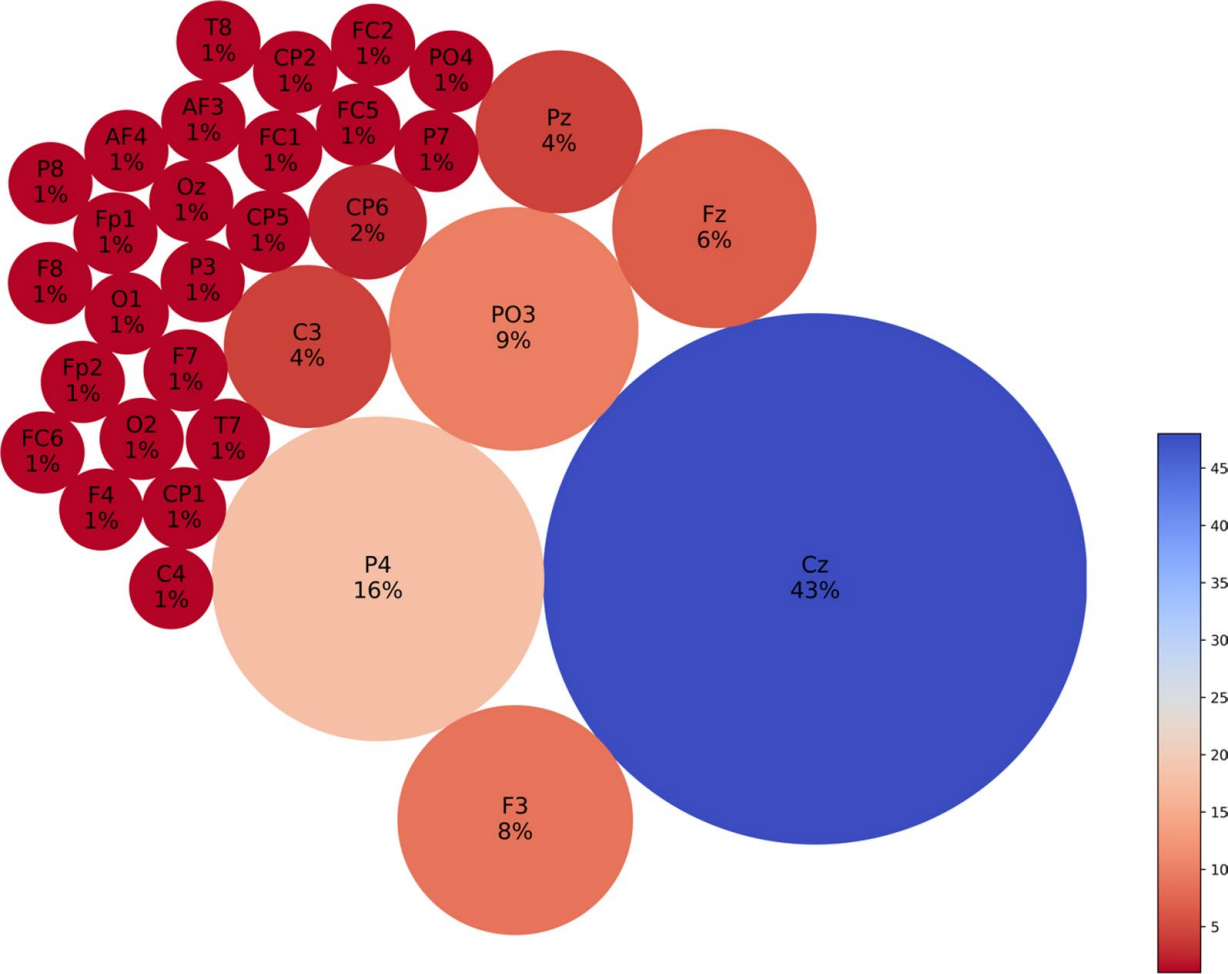
Neuron proportion clustered by connection weights in the subtracted SNNr = SNNr/control – SNNr/cs at resting-state baseline 30–32 s. Blue indicates higher proportion, red indicates less neuron proportion. It shows a larger difference between the CS and control subjects in the brain areas Cz, F3, P4, PO3, Fz, Pz and C3, with a dominant factor of Cz

**Fig. 8:**
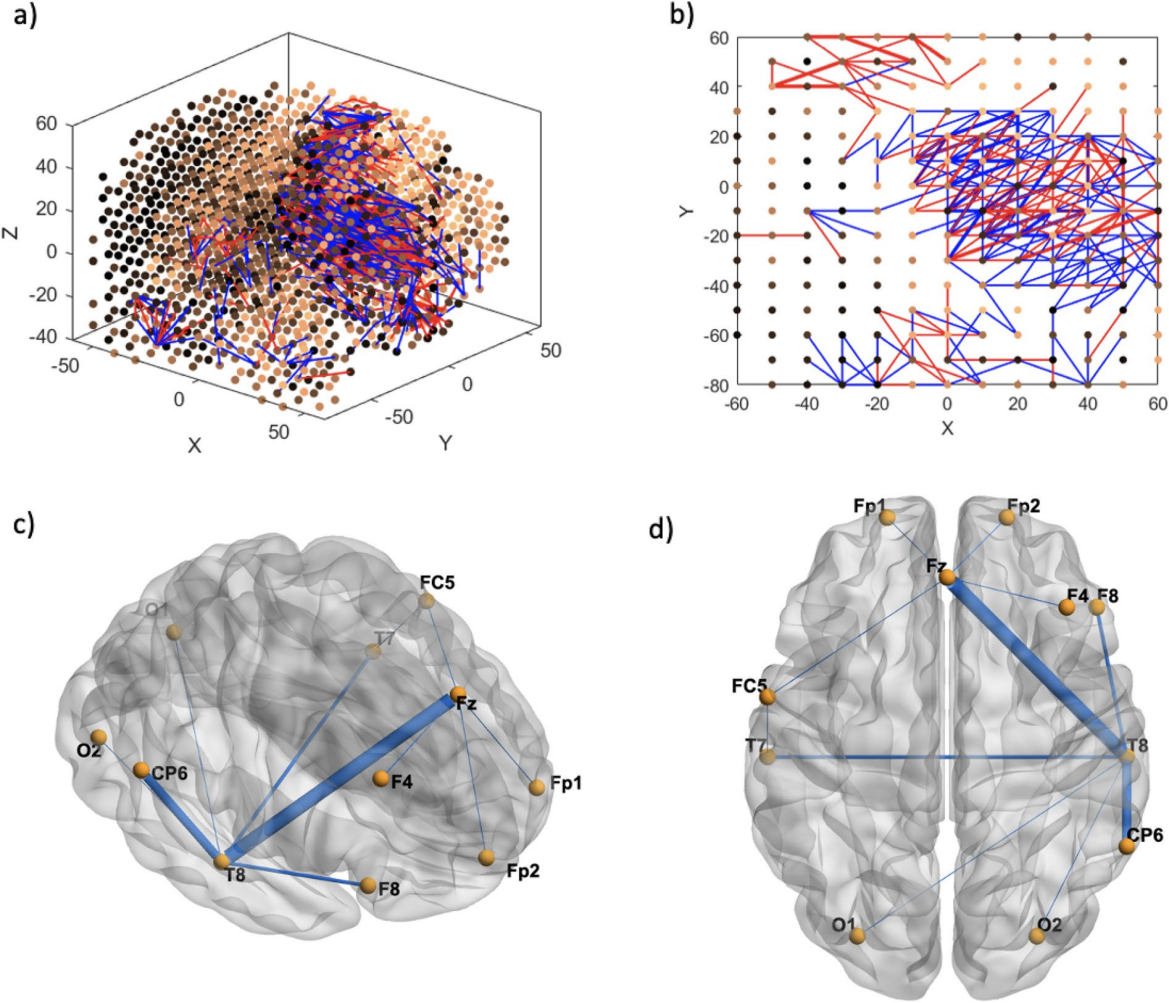
Resting-state baseline 90–92 s subtracted network dynamics. Functional connectivity of neurons is represented by **a** right hemisphere medial view and **b** axial view. Blue lines are positive connections, red lines are negative connections. Brighter neurons have stronger connections. Feature interaction networks between channels are represented by **c** right hemisphere medial view and **d** axial view. Thicker lines indicate a stronger interaction whether they be positive or negative. These interactions confirm our hypothesis that even at the 90–92 baseline, there is a significant difference between the brain information processes of CS versus control subjects

**Fig. 9:**
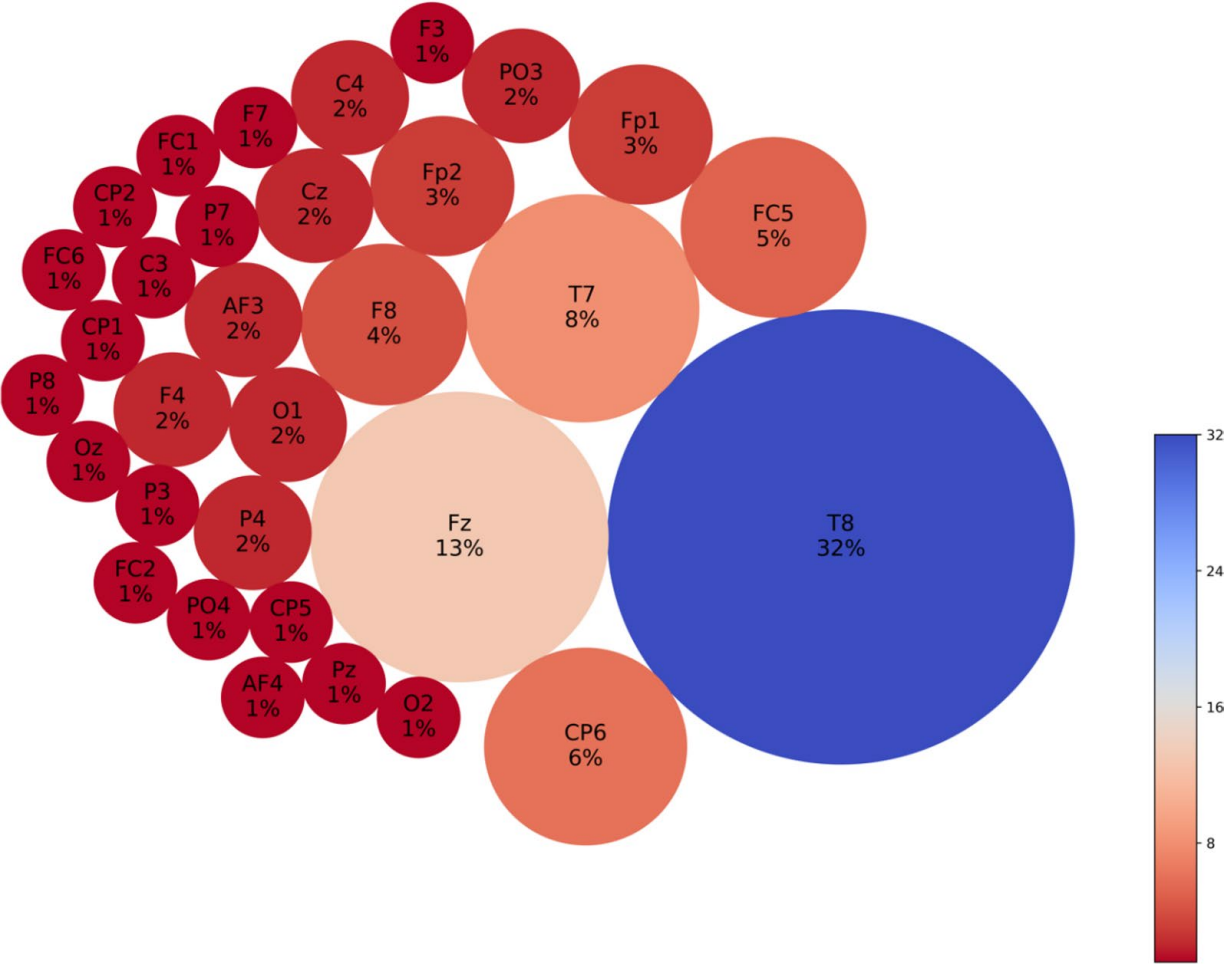
Neuron proportion clustered by connection weight at 90–92 s resting-state baseline. Blue indicates higher proportion, red indicates less neuron proportion. It shows a larger difference between the CS and control subjects in the brain areas T8, CP6, Fz, T7, FC5 with a dominant factor of T8

**Fig. 10:**
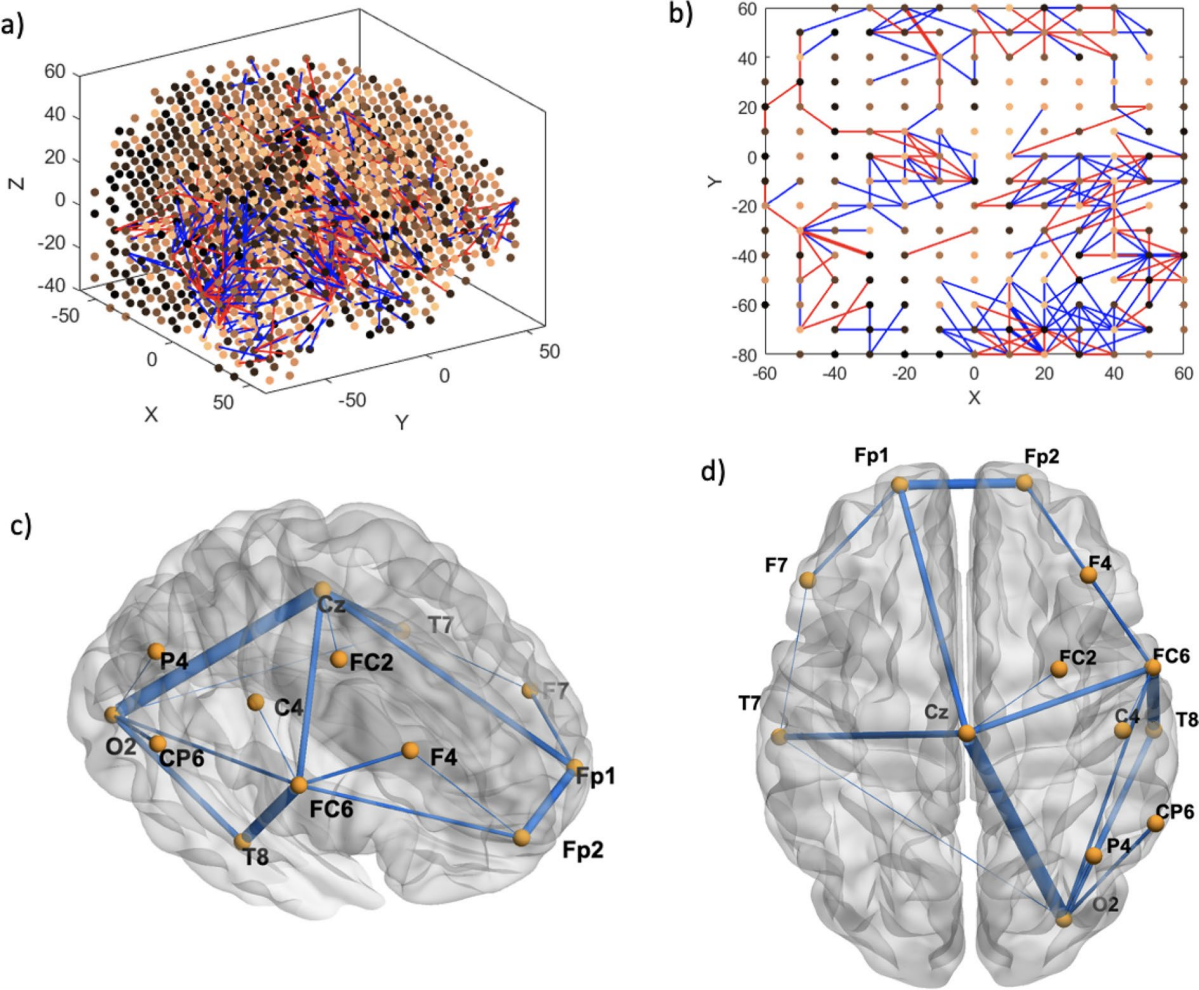
Cybersickness network dynamics of the SNNr = SNNr/control—SNNr/cs, in VR. Functional connectivity of neurons is represented by **a** (medial view) and **b** (axial view). Blue lines are positive connections, red lines are negative connections in **a** and **b**. Brighter neurons have stronger connections. Feature interaction networks between channels are represented by (**c**) and (**d**). Thicker lines indicate stronger interaction whether they be positive or negative. These interactions confirm our hypothesis that there is a significant difference between the brain information processes of CS versus control subjects during the CS manifestation when subjects are exposed to VR. Some of these interactions have been captured already at baseline (see Fig. 6 and Fig. 8)

**Fig. 11:**
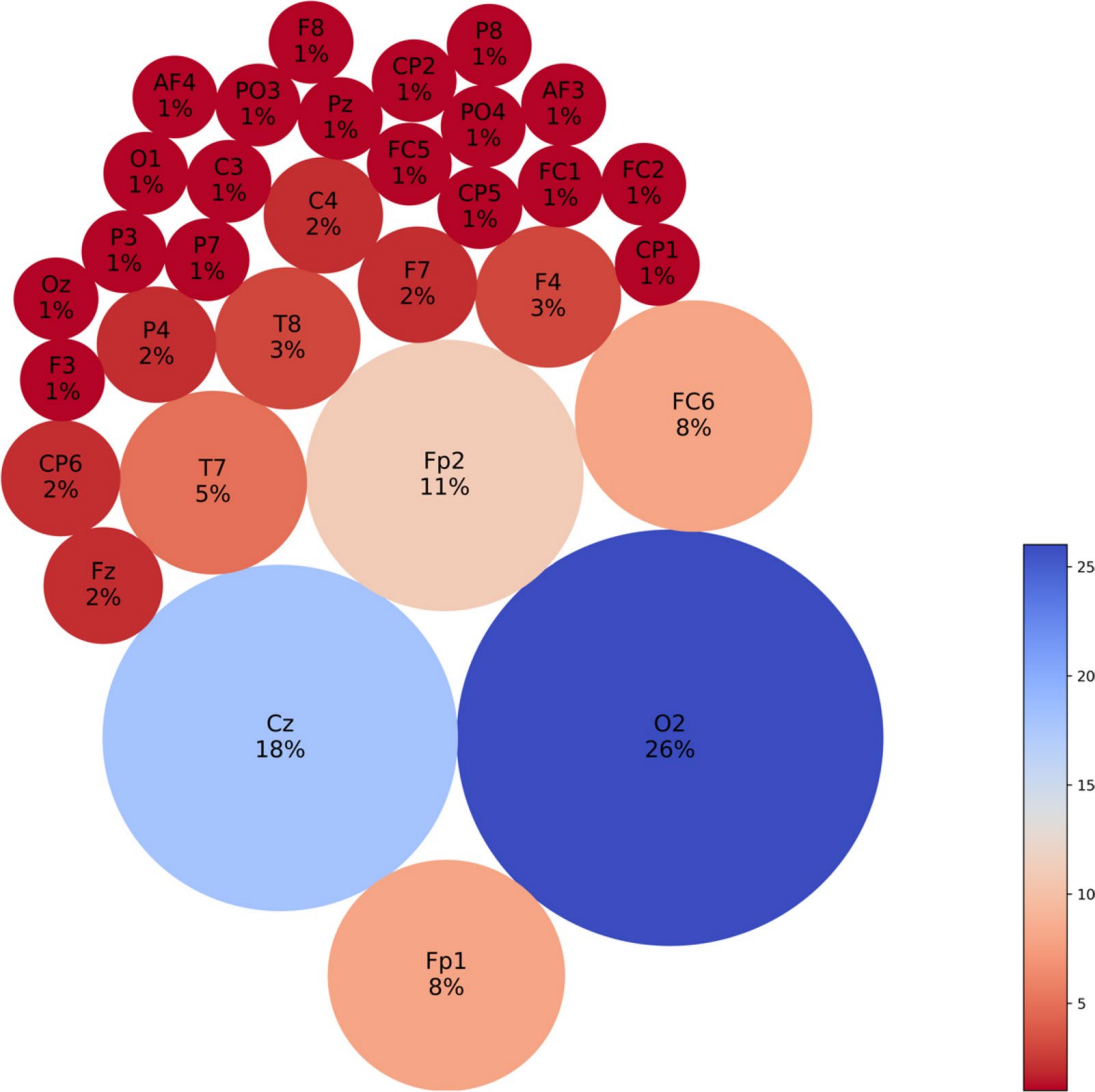
Neuron proportion in SNNr = SNNr/control—SNNr/cs, clustered by connection weights during a VR experiment. Blue indicates a higher proportion, and red indicates a lower proportion of neurons. The difference in the connectivity confirms our hypothesis that there is a significant difference between the brain information processes of CS versus control subjects during the CS manifestation, when subjects are exposed to VR, with dominating brain areas being O2, Cz, Fp2, Fp1 and Fc6. Some of these areas have been captured already at baseline (see Fig. 7)

Functional connectivity analysis at resting baseline EEG (30–32 s) shows that CS prone individuals have more concentrated negative connections in the Cz area, interspersed with surrounding positive connections (Fig. 6a and b), when compared to controls. Feature interaction analysis (FIN) revealed that Cz is likely a hub for brain activity processing in this time segment, either collecting or sending out this information to all the other key channels located in the left and interhemispheric frontal and bilateral parietal areas (Fig. 6c and d). The top 5 features according to neuron proportion were P4, Fz, Cz, PO3 and F3, with Cz being the highest (Fig. 7). A second time segment further on in the baseline (90–92 s) was analyzed, which showed that other important features (T8, CP6, Fz, FC5, T7) can appear at different time segments (Figs. 8, 9). During the CS event, the high functional connectivity seen at baseline in CZ changes to interspersed positive and negative connections. Meanwhile there is a shift towards O2 positive connection dominance. FIN analysis (Fig. 10) showed that both O2 followed by Cz are most likely hubs for cybersickness processing, where both have the highest neuron proportion (Fig. 11). The top 5 features according to neuron proportion were FC6, FP2, FP1, Cz and O2. These results indicate that important areas identified in the baseline that are also found during the manifestation of cybersickness could be important biomarkers of susceptibility to cybersickness.

### Classification results

**Fig. 12:**
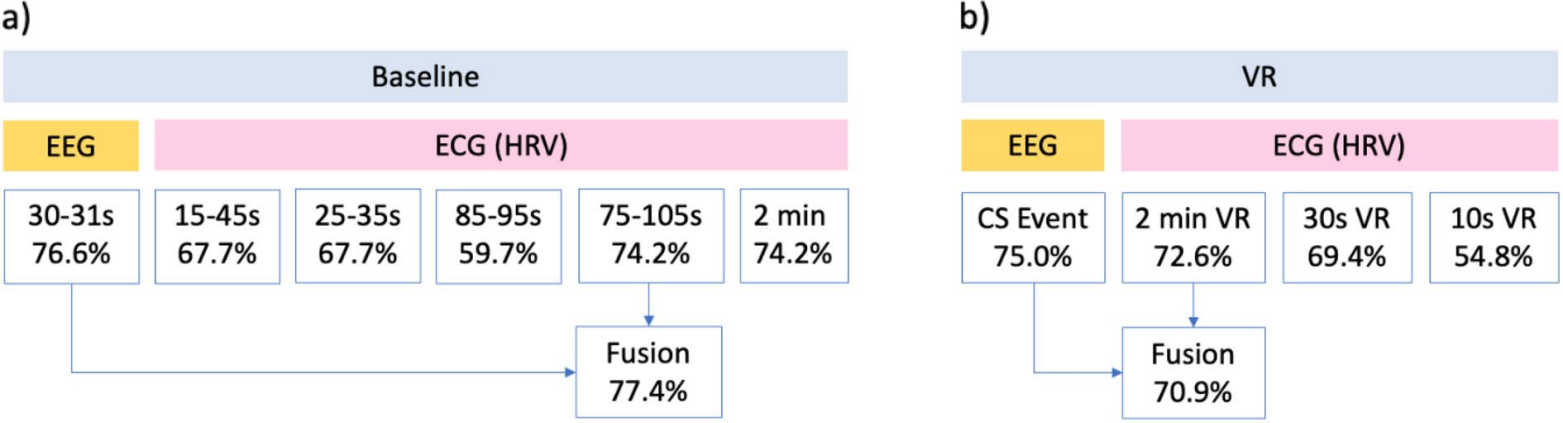
Best KNN classification accuracies for EEG and ECG (HRV) in multiple time segment analyses

Overall, our modified KNN algorithm was the best for both prediction (EEG 76.6%, ECG 74.2%) and detection (EEG 75%, ECG 72.6%) of CS (Tables 1, 2, 3, 4 and 5 and Fig. 12). Both EEG and ECG had similar classification accuracies, although EEG alone was slightly better. Although data fusion of both EEG and ECG could increase accuracy to 77.4% for prediction, it reduced the accuracy for detection to 70.9% (Fig. 12).

**Table 1:** Prediction accuracies of LDA, modified KNN and LGBM classification algorithms at baseline 30–32 s for all subtracted SNNr cubes

Prediction 30–32 s						
I–O connection	32 Channels trained			5 Channels trained		
	LDA	KNN	LGBM	LDA	KNN	LGBM
1471 reservoir + I-O	59.4%	65.60%	68.8%	53.1%	67.20%	53.1%
32	62.5%	67.20%	62.5%	N/A	N/A	N/A
5	54.7%	60.90%	54.7%	59.4%	73.40%	59.4%
P4	48.4%	65.60%	57.8%	46.9%	51.60%	51.6%
Fz	50.0%	59.40%	46.9%	57.8%	70.30%	70.3%
Cz	43.8%	60.90%	57.8%	39.1%	60.90%	54.7%
PO3	0.00%	57.80%	48.4%	0.00%	57.80%	54.7%
F3	53.1%	62.50%	62.5%	53.1%	64.10%	64.1%
Best combo out of 5	Cz + F3 56.3%	P4, Fz, Cz 75.00%	P4, PO3 64%	Cz + F3 62.5%	Fz, Cz **76.6%**	Cz 70.3%

Top accuracies are highlighted in bold

**Table 2:** Prediction accuracies of LDA, modified KNN and LGBM classification algorithms at baseline 90–92 s for all subtracted SNNr cubes

Prediction 90–92 s						
I–O connection	32 Channels trained			5 Channels trained		
	LDA	KNN	LGBM	LDA	KNN	LGBM
1471 reservoir + I–O	53.1%	67.20%	60.9%	50.0%	65.60%	73.4%
32	57.8%	70.30%	64.1%	N/A	N/A	N/A
5	51.6%	68.80%	70.3%	54.7%	64.10%	68.8%
T8	48.4%	71.90%	60.9%	50.0%	62.50%	56.3%
CP6	59.4%	54.70%	56.3%	56.3%	64.10%	67.2%
Fz	50.0%	60.90%	59.4%	0.00%	64.10%	54.7%
FC5	18.8%	60.90%	56.3%	42.2%	60.90%	54.7%
T7	23.4%	65.60%	53.1%	42.2%	57.80%	54.7%
Best combo out of 5	T8 + CP6 + Fz 59.4%	T8 73.40%	T8 61.3%	T8,CP6,Fz,FC5 57.8%	T8, CP6 **75%**	T8,CP6 66.1%

Top accuracies are highlighted in bold

**Table 3:** Detection accuracies of LDA, modified KNN and LGBM classification algorithms at the time of the CS event for all subtracted SNNr cubes

Detection CS onset						
I–O connection	32 Channels trained			5 Channels trained		
	LDA	KNN	LGBM	LDA	KNN	LGBM
1471 reservoir + I–O	57.8%	70.30%	75.0%	56.3%	57.80%	65.6%
32	65.6%	**75.00%**	70.3%	N/A	N/A	N/A
5	50.0%	62.50%	67.2%	53.1%	62.50%	60.1%
FC6	59.4%	65.60%	62.5%	53.1%	67.20%	65.6%
Fp2	42.2%	56.30%	59.4%	43.8%	59.40%	59.4%
Fp1	46.9%	60.90%	57.8%	0.00%	64.10%	59.4%
Cz	25.0%	56.30%	54.7%	53.1%	56.0%	64.1%
O2	51.6%	53.10%	45.3%	0.00%	50.0%	59.4%
Best combo out of 5	FC6 59.4%	Fp2, Cz 68.80%	Fp1,Cz 68.8%	FC6 + Fp2 + Cz 57.8%	Fp2, Cz 68.80%	FC6,Fp1,Cz 68.8%

Top accuracies are highlighted in bold

**Table 4:** Prediction accuracies of LDA, modified KNN and LGBM classification algorithms at different time segments for the best combination of HRV parameters

ECG prediction			
Time segment	ML algorithm		
	LDA	KNN	LGBM
2 Min baseline	56.5% SI + SDNN	**74.2% **PNS + SNS	69.4% SNS
15–45 s	61.3% PNS + SNS + SDNN + RMSSD	67.7% SI	67.7% SNS + SI + RMSSD
75–105 s	62.9% PNS + SNS	**74.2% **SNS	71.0% SNS + SDNN
25–35 s	6.5% RMSSD	67.7% RMSSD	61.3% RMSSD
85–95 s	16.1% RMSSD	59.7% RMSSD	51.6% RMSSD

Top accuracies are highlighted in bold

**Table 5:** Detection accuracies of LDA, modified KNN and LGBM classification algorithms at different time segments for the best combination of HRV parameters

ECG detection			
Time segment	ML algorithm		
	LDA	KNN	LGBM
2 Min VR	61.3% ( PNS or SDNN) + SNS + Mean HR	**72.6% **SNS + SI	69.4% SI + SDNN
30 s VR	66.1% PNS + SNS + SI	69.4% PNS + SNS + SI/ PNS + SI + RMSSD	67.7% SI + SDNN + Mean HR
VR 10 s	56.5% RMSSD	58.1% RMSSD	54.8% RMSSD

Top accuracies are highlighted in bold

### EEG considerations

Some participants were predicted at 30–32 s (samples 3, 4, 18, 24, 27) but not at 90–92 s and vice versa (samples 9, 22 and 23). A hypothesis was that spike count, MSSQ-short percentile scores, SSQ total scores, or CS onset times could explain why some participants were predicted in one baseline segment but not the other. This was not the case, as none of the above showed any deviation from the norm when graphed (Appendix Figs. 13, 14, 15, 16, 17, 18).

It was hypothesized that the spike count at each channel would be different in the CS groups compared to controls at all time segments. We found that the spike count was significantly lower in the CS group than in the controls at the 30–32 s baseline segment (for P4, Fz, Cz PO3, F3) and during VR immersion (O2) (*P* < 0.0001), but not at 90–92 s. Sample 7 in 30–32 s has a high spike count compared to others, as does sample 10 during VR immersion, but removal of these samples does not change the statistical differences (30–32 s *P* < 0.0001, VR immersion *P* < 0.001) between CS and controls spike count. Classification accuracy, however, remains similar for 30–32 s and 90–92 s analysis (76.6% and 75.0%, respectively).

### ECG and HRV considerations

Sympathetic indexes (SNS + SI) outclassed other HRV parameters in terms of classification accuracy. A normal control baseline may be easy to predict or detect, but small changes in these HRV values may not always equate to cybersickness. HRV parameters were useful for classification but there were no significant differences (p>0.05) between CS and Ctrl groups according to a Mann Whitney U rank test. The statistical method may have been limited in its ability to capture relevant differences between groups and this points towards more complex methods of analysis. Furthermore, both of the KNN algorithms employed here employ a min/max voting type on the importance of K-neighbours, which takes into account a weighted Euclidian distance via signal to noise ratio (SNR) between sample data points. These weightings between all data points and between K- neighbours are still influenced by the sample sizes and distribution of the data. Therefore, it may also be possible that a larger sample size is needed to more accurately represent cybersickness.

### Part 2

An extensive analysis was conducted on all 2s, 5s and 10s time segments in the entire baseline recording with 5 channels and 32 channels training. 5 channel trained cubes provided the highest accuracies. The 2 s time segment relating to 110–112 s trained on 5 channels (O1, F8, F7, P8, T7), with only one of I–O connection (F7 channel) used for prediction, yielded the best prediction results of 85.9% accuracy. Overlapping time segments for data lengths 5 s and 10 s did not reach the same performance, achieving a max 75–76% using the best combination of I–O connections. In addition, detection performance was improved to 76.6% after SDSP optimization. Data fusion of this optimised EEG feature vector with ECG HRV parameters provides minimal changes in the ECG accuracy: 77.4% (part 1) versus 74.2% (part 2) for prediction, 70.9% (part 1) vs 72.6% (part 2) for detection (Table 6).

**Table 6:** Improved accuracies for CS prediction and detection using 5 channel EEG trained cubes at all time segments in the entire baseline recording

	EEG	EEG + ECG fusion
Prediction	**85.9%** (F7, 110-112s I-O connection)	74.2% (75–105 s, SNS)
Detection	76.6% (FP1, Cz I-O connection)	72.6% (2 min, SNS + SI)

Top accuracies are highlighted in bold

Analysis included time segment and data length optimization (prediction only), and SDSP optimization of the *mod*, *driftup* and *driftdown* parameter. Prediction used modified KNN algorithm. EEG trained on top 5 channels. Neuron proportions at 110-112s: O1 (9%), F8 (9%), F7 (7%), P8 (7%), T7 (6%).  Fusion accuracies increased for detection but not for prediction

## Discussion

### Classification

This paper presents a proof of concept for on-the-spot prediction of cybersickness at resting state baseline and near-instant detection of cybersickness during its onset. The algorithms are based on brain inspired SNN architectures and HRV classification. Another study has also demonstrated the predictive capacity of their algorithm for CS at resting baseline with a smaller sample size of *n* = 19 [27]. Near-instant detection was demonstrated by Nam et al. [28] but required PCA preprocessing, power spectral analysis for EEG and 7 other biosignals. The present study shows that only 2 s of EEG data and 30 s of ECG data are required, and both biosignals can be used individually or together to predict and detect CS. The modified deSNN-KNN classification algorithm produced the best results in terms of accuracy, over LDA and light-GBM. It was found that similar classification accuracies can be obtained using either earlier (30–32 s, 76.6%) or later time segments (90–92 s, 75%) at baseline. Upon further investigation, the study found that time segment optimization was still important (85.9%). Simplifying feature vectors by removing reservoir—output neuron connections, and leaving the direct connections of input neurons to output neurons increases accuracies (Tables 1, 2 and 3). In addition, reducing redundancy in training data by focusing on key cybersickness relevant areas also has the same positive effect on accuracy. However, in the case where a model is trained on all 32 features, but only the top 5 features are considered, a reduction leads to a decrease in accuracy (75.00–68.80%) (Table 3). This highlights that in idealistic scenarios, not just a few but all features a model is trained on should be considered when eliminating redundancy. However, it is important to note that there is a trade-off in considering all features, as computational cost increases when conducting exhaustive searches.

Our analysis did not reveal why some participants were predicted in one baseline segment but not the other. An explanation is that this could be due to differences in the temporal characteristics of the spiking activity of neurons captured by the connection weights between input clusters and between individual reservoir neurons. Another explanation could be due to the nature of clinical studies, where there is interindividual variation between participants.

Fusion of EEG and ECG did not yield much improvement in accuracy, and in the case of detection it worsened accuracies in part 1. Multi-modal data fusion was investigated to explore if information from two organs would lead to increased accuracy, especially because they are biologically linked through the nervous system both in anatomy and also in association to nausea [29]. Because of the disparity in classification performances between EEG and ECG, it is likely that the classification algorithm’s ability to differentiate strongly between labels is ‘drowned’ out by the ECG HRV features, which is why the EEG now adds no useful information for classification beyond what is already there. It is also possible that KNN being a distance-based algorithm, gets worse with higher dimensional feature vectors, a trend shown as well in the improved classification performances the less features there are in the feature vector.

### MSSQ and SSQ scores

In our experiment, the MSSQ-short was not a good predictor of cybersickness induction or sickness ratings. This points towards the need for questionnaires more targeted at visually induced motion sickness [30] to assess susceptibility. SSQ scores were a good adjunct to the subjective cybersickness reports in the separation of cybersick and control groups.

### Related spatiotemporal brain dynamics were discovered in the following areas

Fz Brodmann 8 visual attention and eye movements.

T8, T7: Auditory processing.

CP6: Auditory processing, speech comprehension.

O1, O2: Retinotopic mapping of visual scene, edge detection.

P4: Angular gyrus attention, memory retrieval, language number processing, spatial cognition.

PO3: Associative visual cortex (V3, V4, V5).

F3: Frontal eye fields, visual attention and eye movements.

FC5, FC6: Brocas speech production and articulation (primarily left hemisphere), language processing.

FP1, FP2: Executive function, decision making.

F7, F8: Active maintenance of stimulus information, interoceptive, limbic emotion-motivational, and sensory input integration.

CS is a complex condition with many brain areas involved [31, 32]. Presented in this study is functional connectivity of the brain that predicts future CS, meaning that an individual with similar neural maps may be susceptible to cybersickness, and connectivity that marks the CS event. In the present study, a high neuron proportion grouped by connection weight of frontal (FC6, FP1, FP2) regions during the CS event, and temporal regions (T8) during resting baseline are consistent with another study showing changes in these areas well into the CS event. In addition, areas involved in CS include those for visual + attention processing and executive function (CP6, O2, PO3, F3, F4, FP1, FP2). Liu et al. [31] found reduced gravitational frequency means (transition of EEG power spectral density, temporal changes within a frequency band), and gravitational frequency standard deviation (dispersion of brain signal) at FP1, FP2, TP9 and TP1. Power spectral entropy (disorder of time sequence signals and irregularity of multi-frequency component signals) and Kolmogorov complexity (time domain complexity) were all reduced at FP1 and FP2 during VIMS [31]. However, it was noted that these changes may be related to other factors, such as alertness level or various mental conditions, and not limited or specific to VIMS. Our finding of an increase in O2’s interaction with other areas during cybersickness highlights that visual processing is altered beyond just the demands of normal visual processing in VR. O2 has been selected as an important feature in other machine learning studies as well [28, 33, 34], but the possible differences in results compared to the discussed brain analysis and imaging studies may be in the temporal specificity (2 s long) of our analysis compared to longer data lengths analysed.

Of interest is the brain activity hub found at Cz, which had altered connectivity at resting-state baseline as well as during the onset of cybersickness when compared with controls. Reduced spike count at Cz before VR immersion may indicate that there is less frequency of communication from this area to other connected areas. Cz interacts with three cortices simultaneously, the somatosensory, motor and also is positioned over the mid cingulate, which has increased functional connectivity with the left V5/MT during cybersickness [35]. Krokos, Varshney [36] found high activity power in the central regions similar to the location of Cz, of average scalp maps according to independent component analysis. Brodmann area 5 corresponds to Cz, which is part of the superior parietal lobule and post central gyrus. It is located immediately posterior to the primary somatosensory cortex. Neuroimaging evidence suggests that this area contributes to movement planning. Furthermore, one study showed a correlation between the activity of area 5 neurons and the starting or final coordinates of limb movement. This suggested that BA5 is involved in processing spatial information for limb movement. Emerging evidence suggests that BA5 is also involved in the inhibition of movement [37]. A transcranial magnetic stimulation study found a causal role for BA5 in the regulation of corticospinal output during preparation that differentiates between whether a movement is withheld or executed [38]. Cz’s role in movement and also as a marker of future cybersickness at resting baseline lends possible credence to the postural instability theory of motion sickness, which postulates that postural instability is both a marker and a predictor of motion sickness, likely extending as well to cybersickness in virtual reality [39]. Although our results suggest that processes related to motor control are altered during the event, further research is still required to link the brain dynamics related to postural instability and cybersickness. Furthermore, a recent study shows that postural instability itself is not a good predictor of cybersickness [40]. For purely visually induced motion sickness (VIMS), increases in functional connectivity were also found between the right MT/V5 and anterior insula. Decreased functional connectivity was also found between the left and right V1 [35]. The left MT/V5 in particular is an area important for processing of “what” but not “where”, in priming for motion direction but not spatial position [41]. Nonetheless, cortical areas that control movement and visual processing are clearly involved in cybersickness.

Interestingly, cortical areas for visually induced cybersickness also overlap with areas involved in vestibular processing: Cz and FC6—premotor and supplementary motor (movement processing, planning and inhibition) and P4—medial superior temporal (motion detection). In this study, it can be observed that the size of the nodal cluster and strength of connectivity shift to right hemispheric dominance during CS, a preference also observed in vestibular processing. Overall, there appears to be an alteration of activity and connection in areas related to motor control and planning, as well as visual processing. These areas may become targets of intervention for future studies [29].

F7 was highlighted as an area of interest after its correlation as an input to produce high accuracies in part 2 of the analysis. F7 relates to Brodmann area 45, the inferior frontal gyrus (IFG) [42]. The IFG and also anterior insular (AI), which also has associations with V5/MT as described above, is part of the ventrolateral prefrontal cortex (VLPFC). The VLPFC is involved in a host of functions related to active maintenance of stimulus information, including being both a control and integrative node in the brain and an interface between sensory and motor areas [29, 42]. Not only does it handle awareness of the immediate moment but also implementation of reactions to it. Furthermore, it is involved in forming immediate connections between sensory processing and action control [43]. In addition, F7 integrates interoceptive, limbic emotion-motivational (from orbitofrontal and subcortical areas), and sensory input (object identity from the ventral visual pathway) [43, 44-46]. In particular, visual information of behavioural significance travels from the ventral pathway to the VLPFC, and later to the dorsolateral prefrontal cortex (DLPFC) and arcuate area. From here additional information from the dorsal pathway is then integrated to form a precursor of motor command [44]. In a transcranial magnetic stimulation (TMS) study, it was found that the left VLPFC had a role in the regulation of negative emotions using positive reappraisal, which is the ability to reinterpret the meaning of an emotional event or stimulus into a more positive light. The VLPFC further produces a top-down biasing effect [47] that drives selection and retrieval dynamics in the posterior cortex [43, 45-48]. There also exists underlying asymmetry in the activation of the IFG/AI. F7 refers to the left IFG, and it has been found that incongruency in a flanker task activates IFG/AI, whereas the right IFG/AI (F8 was also a top 5 feature along with F7 in the best time segment) is activated more by errors [49]. The IFG/AI is also involved in post error slowing, where performance is slowed down due to making an error [29, 43]. The IFG/AI-anterior cingulate cortex network is also thought to be involved in incongruency detection and resolving, and the ability to inhibit inappropriate responses [43]. All together, it is not too far a stretch to imagine that a brain area involved in immediate recognition, regulation, resolution and action on the incongruency and error in the environment could be one of the key role players in susceptibility to cybersickness, and this is reflected in its superior performance for prediction amongst all other features. The additional discovery of F7 in part 2 of the analysis has led to a comprehensive picture of cybersickness, in which there is now a node specific in function for integration and control in response to incongruent environmental information commonly found in VR stimuli that induce cybersickness [50], in addition to areas mention above involved with visual processing (O2) and motor planning (Cz).

### ECG

This study tried to use ultra-short-term RMSSD recordings in an attempt to classify cybersickness without having to capture more than 10 s of ECG data. Ultra-short-term RMSSD recordings (30 s and 10 s) have been statistically reliable in previous studies, but this parameter alone does not yield high accuracies (Tables 4 and 5). Although reductions in RMSSD have been associated with cybersickness intensity, more evidence is needed to explore the role of parasympathetic cardiac indicators in cybersickness [51]. Conversely, nausea and visually induced motion sickness have been found to be mediated by the brain with links to sympathetic cardiac responses [29, 35, 52-54]. Although statistical differences between HRV parameters were not found, it was found that classification algorithms for cybersickness using sympathetic HRV indexes are still viable. This finding is shared with other studies where HRV has shown promise for cybersickness classification [4]. This suggests that the differences in sympathetic parameters of HRV in cybersick people versus control are more complex and simpler types of statistical analysis may not capture this complexity.

### Future suggestions and limitations

Given that HRV is computed using R–R intervals of an ECG wave, it may be the case that other parameters, arising also from other aspects of the ECG wave could be helpful as features, such as those used in detecting other pathologies like atrial fibrillation [55-57]. Further research could elucidate on this matter.

The NeuCube SNNr has some similarities to a liquid state machine (LSM) [58]. In a LSM, both reservoir computing [59] and a spiking neural network is used to learn dynamical systems. Spike inputs cause a propagation of spike activity throughout the reservoir, which are like ‘ripples’ caused by a ‘stone falling into liquid’. However, NeuCube differs in that the structure is brain-inspired with stationary spatial mapping of inputs, and in that it uses unsupervised and supervised learning [60]. This application of SNNr allowed for new knowledge generation about CS and directed feature selection, and even revealed promising targets for intervention. Still, the reservoir and output layer connections were detrimental to classification performance. It is likely that these connections served as noise to the classified feature vector. However, the information synthesized and stored within the SNNr is still meaningful and valuable. Other training parameters of the cube could be optimized such as the leak rate in membrane potential, the learning rate, refractory time for neuron firing and number of training iterations [6]. Nonetheless this points towards the need for future research on how to maximize interpretability and knowledge discovery alongside classification performance. Moreover, given that SNNr activity is primarily influenced by its initial connections, a careful consideration on how to initialize neurons within the SNNr is needed. In this study, the neurons within the SNNr have no distant connections because of the limited radius set by the small world connectivity approach. However, in an actual brain, there are both distant and local connections between neurons [61]. NeuCube allows for long distance connections to be created through a probability [6], but these connections are not currently biologically informed. Future research can expand on how to generate a more biologically plausible SNNr and on how to use the information generated within it to enhance model performance.

Some additional points also require consideration. This study used machine learning to extract information about the spatiotemporal processes within the cybersick brain but future studies could explore the role of the interplay between motor control, motor planning and visual processing in VR on CS. Although, all the EEG channel signals are inputted back into the spiking neural network in a spatially locked manner (to the same talairach coordinate), it is worth to note that, the present study also have the EEG’s well-known limited spatial resolution. The feature interaction network analysis only showed interactions between cortical areas, but not whether they were increasing or decreasing connections. Future studies could shed light on how key cybersickness centers in the brain act to control the flow of information between cortical areas. Furthermore, the finding that different features can be found at different time segments, but still give similar accuracies, points towards the complexity of the cybersickness condition within the brain. It may therefore be of interest to look at the change in features over time, rather than the features at snapshots in time to understand cybersickness in more detail. Finally, it is not yet known if the multimodal data fusion shown in this study could be improved by other biosignals and this could be valuable research to conduct moving forwards.

## Conclusion

The paper proposes and demonstrates that a brain-inspired spiking neural network (SNN) model can be created and used for on-the-spot prediction of cybersickness at resting state baseline and near-instant detection of cybersickness during its onset. Using this SNN model means that instead of storing raw data, each sample can be stored as a feature vector representing brain activity, which means less memory storage and processing requirements. The model can be dynamically updated on new data, modifying both the weighted template neural map and the feature vectors to produce new insights. HRV alone or data fusion with EEG are useful biosignals for the prediction and detection. Motor processing areas under Cz, visual processing areas at O2, as well as control and integration of incongruent information at F7 are key sites for CS. These sites contain biomarkers as a precursor and detector of cybersickness and could be useful target areas for clinical intervention.

## Figures and Tables

**Fig. 13:**
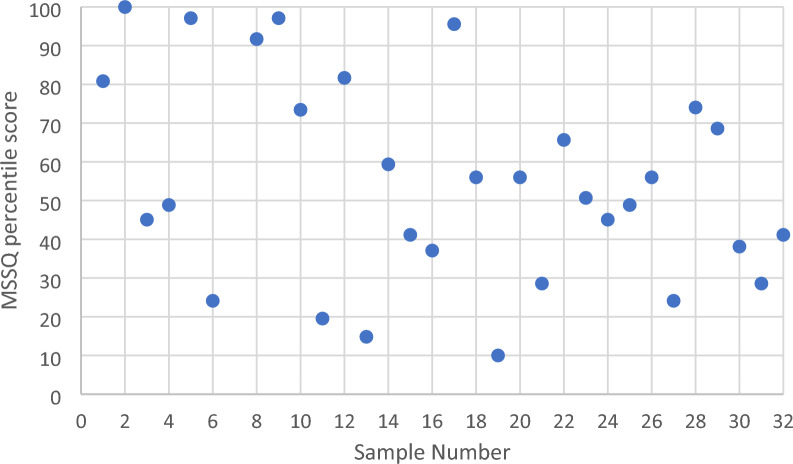
MSSQ percentile score breakdown for the CS group

**Fig. 14:**
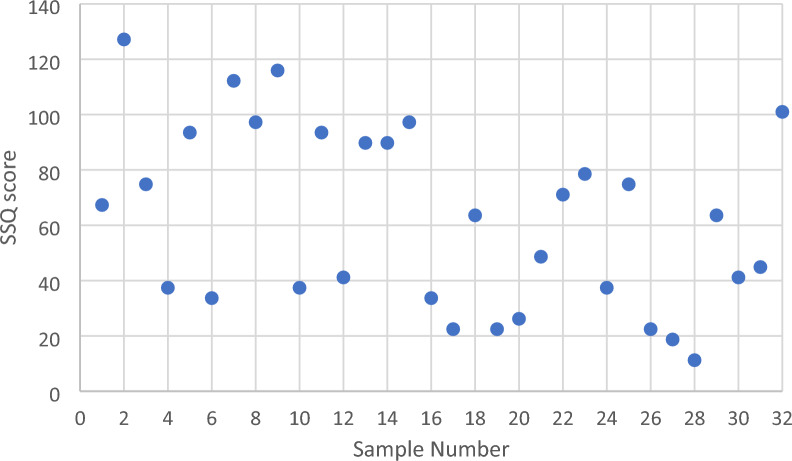
SSQ score breakdown for the CS group

**Fig. 15:**
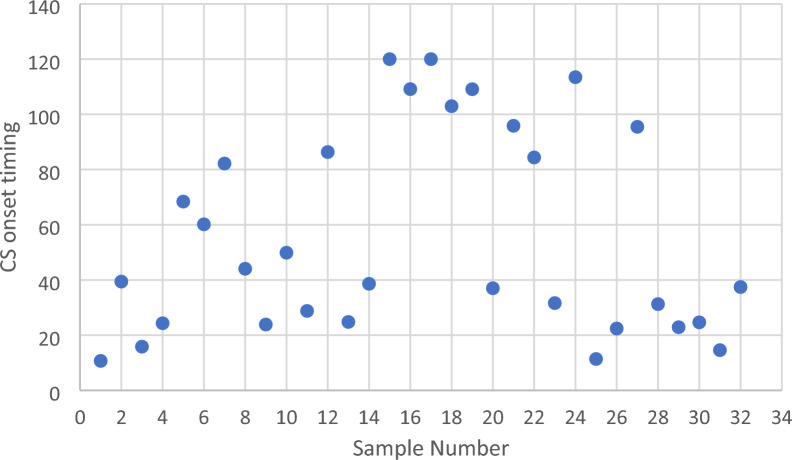
CS onset timings (seconds)

**Fig. 16:**
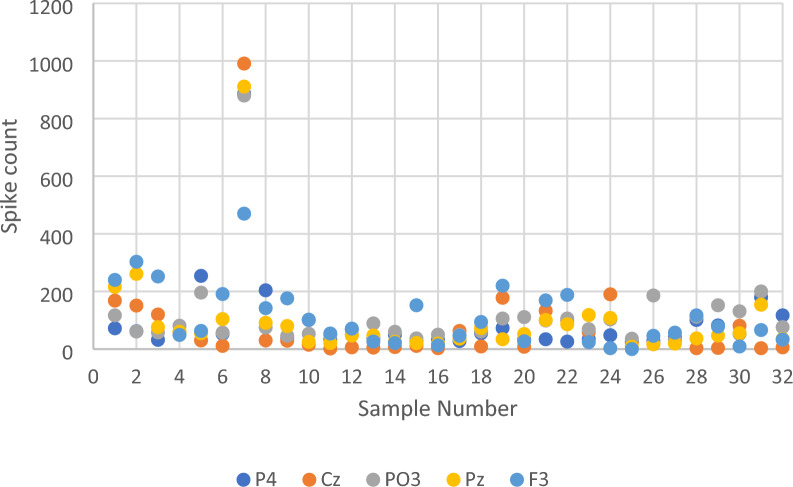
Spike count of 30–32 s time segment for all CS participants. Sample number 3, 4, 18, 24 and 27 were predicted at 30–32 s but not at 90–92 s

**Fig.17:**
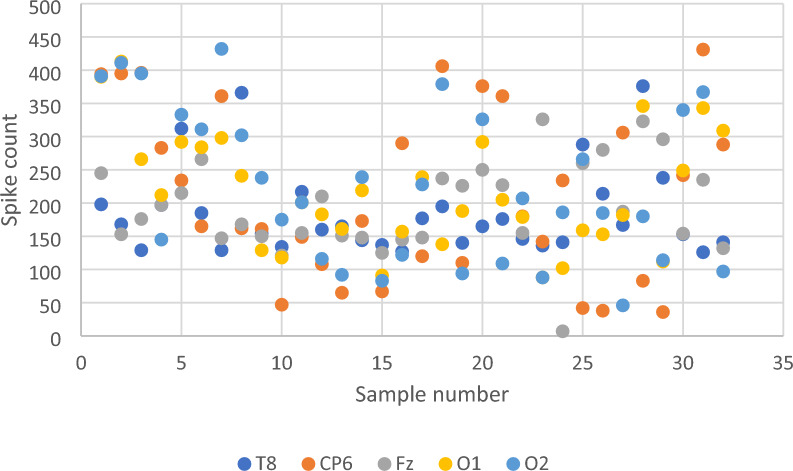
Spike count of 90–92 s time segment for all CS participants. Sample number 9, 22, 23 were predicted at 90–92 s but not at 30–32 s

**Fig. 18:**
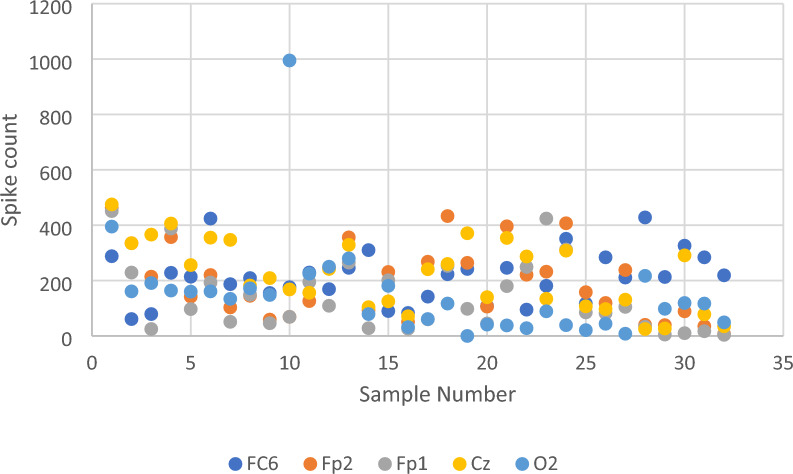
Spike count for all CS participants during the CS onset

## Data Availability

The data sets analysed during the current study are available from the corresponding author on reasonable request.

## Appendix

See in Figs. 13, 14, 15, 16, 17, 18.

## Abbreviations

VR: Virtual reality
CS: Cybersickness
ECG: Electrocardiogram
EEG: Electroencephalogram
SNN: Spiking neural network
HRV: Heart rate variability
KNN: K-nearest neighbor
LL: Left leg
RL: Right leg
LA: Left arm
RA: Right arm
MSSQ: Motion sickness susceptibility questionnaire
SSQ: Simulator sickness questionnaire
PNS: Parasympathetic nervous system index
SNS: Sympathetic nervous system index
SI: Stress index
SDNN: Standard deviation of N–N intervals
RMSSD: Root mean squared of successive differences
STDP: Spike timing dependent plasticity
SDSP: Spike driven synaptic plasticity
deSNN: Dynamic evolving spiking neural network
SF: Step forward
LSM: Liquid State Machine
SNNr: Spiking neural network reservoir
FIN: Feature interaction network
RO: Rank-order learning rule
LOOCV: Leave-one-out cross validation
SNR: Signal to noise ratio
LDA: Linear discriminant analysis
LightGBM: Light gradient boosting machine
GBDT: Gradient boosting decision tree
Goss: Gradient-based one-side sampling
Dart: Dropouts meet multiple additive regression trees
VIMS: Visually induced motion sickness
VLPFC: Ventrolateral prefrontal cortex
DLPFC: Dorsolateral prefrontal cortex
